# Compression Molding Flow Behavior and Void Optimization of an Integrated Circuit Package with Shielding-Metal-Frame

**DOI:** 10.3390/polym17101301

**Published:** 2025-05-09

**Authors:** Ting-Yu Lee, Yu-Li Chen, Sheng-Jye Hwang, Wei-Lun Cheng, Chun-Yu Ko

**Affiliations:** 1 Department of Mechanical Engineering, National Cheng Kung University, Tainan 701, Taiwan; 2 Advanced Semiconductor Engineering, Inc., Kaohsiung 811, Taiwan

**Keywords:** system-in-package, heterogeneous integration, epoxy molding compounds, air taps, Taguchi methods

## Abstract

As the demand for smaller and more multifunctional integrated circuit (IC) products increases, system-in-package (SiP) has emerged as a key trend in IC encapsulation. However, the use of polymer-based materials such as epoxy molding compounds (EMCs) introduces complex flow behaviors during the encapsulation process, often leading to void formation, especially in highly integrated SiP structures. This study employs the Moldex3D 2024 R3 simulation software to perform mold-filling analyses of SiP packages using EMC as the encapsulant. The objective is to investigate why voids are consistently observed in the leftmost column of the strip and to determine how to reduce the void size using the Taguchi optimization method. To replicate the actual vacuum-assisted molding conditions, a 1/5 strip model with venting was established. Results show that the flow dynamics of the polymeric encapsulant are significantly affected by shielding frame geometry. Among various design modifications, adding an additional column of shielding metal frame on the left side of the strip most effectively reduces void formation. This research highlights the importance of polymer flow behavior in void prediction and optimization for advanced SiP packaging, providing practical guidelines for material-driven design improvements in IC encapsulation processes.

## 1. Introduction

As the demand for smaller and more multifunctional integrated circuit (IC) products grows, system-in-package (SiP) has emerged as a key development trend in IC encapsulation, and SiP technology offers significant advantages in integration and miniaturization, making it a key solution in modern electronic product design. By integrating multiple functional chips and passive components into a single package, SiP effectively reduces the system size and supports heterogeneous integration. The SiP packaging structure studied in this paper includes a die, passive components, and shielding metal frames. Compared to transfer molding, compression molding offers a shorter filling distance and lower filling pressure, making it more suitable for multi-component packaging. This study focuses on addressing void issues in the compression molding process for SiP.

Masanori et al. [1] highlighted that, compared to transfer molding, compression molding reduces the waste of epoxy molding compounds (EMCs) during the filling process. Sharma et al. [2] demonstrated the effectiveness of compression molding for molded underfill (MUF), noting that no voids were detected. The rheological properties of EMC significantly affect the filling process, as rheology studies the flow and deformation of materials. In this study, the cross Castro–Macosko model is applied to describe the viscosity and flow of EMC, considering its curing behavior as a thermosetting material. Abdullah et al. [3] confirmed the reliability of the cross Castro–Macosko model for EMC rheology. Bidstrup-Allen et al. [4] used Kamal’s model to simulate the degree of EMC cure over time and temperature, while also employing the Castro–Macosko model to track viscosity changes during filling.

Hwang and Chang [5] introduced a P–V–T–C model to describe the volume shrinkage of EMC under isothermal and isobaric conditions, enabling predictions of warpage and residual stress after molding. In a subsequent study, Hwang and Chang [6,7] further explored isobaric cure shrinkage behaviors under varying temperatures and pressures, emphasizing the significant impact of cure-induced shrinkage on the package warpage base on Hwang and Chang. Chang et al. [8] developed a comprehensive 3D numerical model to simulate mold-filling behavior, which effectively predicts melt flow patterns and captures melt front dynamics. Teng and Hwang [9] expanded the use of the P–V–T–C equation to forecast process-induced warpage in electronic packages, demonstrating the benefits of incorporating both thermal and cure shrinkage into the model and employing the Taguchi method to optimize the process parameters.

Void formation is a critical concern in the IC encapsulation process. Chae [10], Choi [11], and Khor [12] identified that voids typically result from instability at the EMC melt front. Tamil et al. [13] used Moldex3D to predict void formation locations in MUF and applied a design of experiments (DOE) approach to optimize process parameters, thereby reducing the void size. Khor et al. [12] also noted that moisture trapped in voids can vaporize during heating, potentially leading to a popcorn effect during reflow [14]. Additionally, Chang and Hwang [7] studied the interaction between EMC and mold surfaces, developing a mold adhesion force tester to quantify adhesion during the packaging process. This device provides valuable insights into how adhesion impacts the quality and reliability of IC packaging, particularly in reducing the defects related to mold adhesion.

Liu, Zhang, and Yang et al. [15,16,17,18,19] approached the problem from a material design perspective, investigating the microstructure of high-defect-density composites. Their concepts regarding defect engineering and interfacial control provide valuable insights into the modeling of viscosity and flow behavior in polymer encapsulation materials. Such microstructural optimization strategies highlight the critical role of structural design in tuning overall material performance, offering potential reference value for future packaging simulations and process parameter optimization.

Through a comprehensive literature review, we found that relatively few studies have specifically focused on void formation in SiP packaging, especially in the context of heterogeneous integration. Even fewer works have attempted the predictive simulation of void behavior using polymer-based materials such as epoxy molding compounds (EMCs). The reviewed literature, however, provides valuable insights into how previous researchers have modeled the flow behavior of EMCs and investigated the potential causes of void formation.

## 2. Flow Chart of Simulation Process

In this chapter, the Moldex3D 2024 R3 simulation software and the geometry of the product are first introduced. Next, the actual problems of the product are explained. Then, the models, material parameters, and boundary conditions used in the simulation are described. Finally, the simulation results are presented and analyzed.

**Figure 1:**
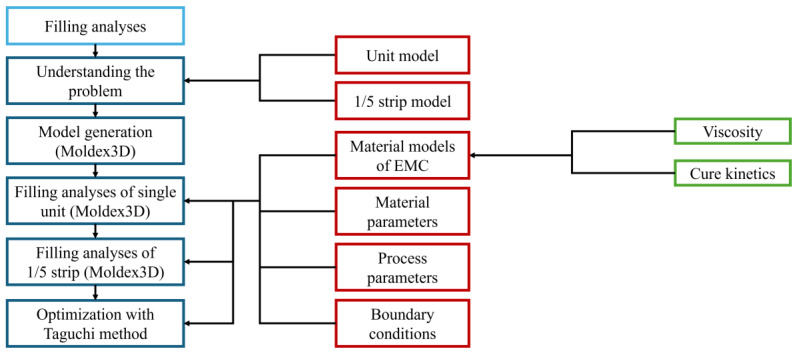
Flow chart of the simulation process.

The flow chart of the simulation process is presented in Figure 1. First, the actual problems of the product are understood, and then, the mesh models are established. Next, the materials, parameters, and process parameters are input into the software for simulation. Finally, the Taguchi method is used to analyze and find the optimal solution.

## 3. Numerical Simulations

### 3.1. Mold Flow Analysis Theorem

The flow of EMC is described by the three main governing equations of fluid mechanics.

#### 3.1.1. Continuity Equation

The continuity equation is used to describe the mass conservation of EMC flow which is based on control volume calculations. The equation is shown below:

(1) $$\frac{\partial \rho }{\partial t}+\nabla \cdot \left(\rho \overset{⃑}{V}\right)=0$$

where ρ: density of EMC; $\overset{⃑}{V}$: velocity; t: time; ∇: divergence operator.

#### 3.1.2. Momentum Equations

The momentum equations are used to describe the variation in momentum of fluid within a control volume. The equations are shown below:

(2) $$\rho \left(\frac{\partial \overset{⃑}{V}}{\partial t}+\overset{⃑}{V}\cdot \nabla \overset{⃑}{V}\right)=\nabla \cdot {\overset{⃑}{\overset{⃑}{\sigma }}}_{\mathrm{total}}+\rho \overset{⃑}{g}$$

(3) $${\overset{⃑}{\overset{⃑}{\sigma }}}_{\mathrm{total}}=-P\overset{⃑}{\overset{⃑}{\delta }}+\overset{⃑}{\overset{⃑}{\tau }}$$

where ∇: gradient operator; $\overset{⃑}{V}$: velocity; P: pressure, $P\overset{⃑}{\overset{⃑}{\delta }}$ is hydrostatic pressure; ${\overset{⃑}{\overset{⃑}{\sigma }}}_{\mathrm{total}}$: total stress; $\overset{⃑}{\overset{⃑}{\tau }}$: extra stress; $\overset{⃑}{g}$: gravitational acceleration.

#### 3.1.3. Energy Equation

The energy equation, based on the principle of energy conservation, is used to describe the energy variation in EMC due to curing reactions, heat conductivity, and viscous dissipation effects. The equations are shown below:

(4) $$\rho C_{p}\left(\frac{\partial \overset{⃑}{V}}{\partial t}+\overset{⃑}{V}\cdot \nabla T\right)=\nabla \cdot \left(k\nabla T\right)+(\overset{⃑}{\overset{⃑}{\tau }}:\nabla \overset{⃑}{V})+\dot{\alpha }\Delta H$$

where $C_{p}$: specific heat of EMC; T: temperature of EMC; k: heat conductivity; ΔH: curing reaction heat; $\dot{\alpha }$: curing rate of EMC.

#### 3.1.4. Taguchi Method

Taguchi method was invented by Dr. Taguchi. A large amount of data can be obtained with fewer experiments or simulations using the Taguchi method [20]. The following steps are the experiment design process in the Taguchi method:

1. Define quality characteristics.
2. Set the control factors and their levels.
3. Design the orthogonal array.
4. Conduct the simulations or experiments.
5. Analyze the data of the results.
6. Determine the optimal combination of control factors.
7. Validate by the simulations or experiments.

In the Taguchi method, the lower the quality loss of the product, the higher the quality. The closer the quality characteristic is to the target value, the smaller the quality loss. When there are n products, the average quality loss is expressed by the following equations:

(5) $$Q=k\frac{\sum_{i=1}^{n}{\left(y_{i}-m\right)}^{2}}{n}=k[\mathrm{MSD}]$$

(6) $$Q\;=\;k\frac{\sum_{i=1}^{n}{(y_{i}-m)}^{2}}{n}\;=k[{(\bar{y}-m)}^{2}+{\;S}^{2}]$$

Q: average quality loss; n: number of products; $y_{i}$: quality characteristics of the i-th product; m: target value of the quality characteristics; and MSD: mean square deviation; ${(\bar{y}-m)}^{2}$: The deviation from the center value; $S^{2}$: The standard deviation.

In this study, the smaller the quality characteristic that is adopted when the target value m is zero, the better. It can be expressed as the following equation:

(7) $$\mathrm{MSD}={\bar{y}}^{2}+{\;S}^{2}$$

When implementing the Taguchi method, it is necessary to set control factors and their level, which are the operational variables in the experiments or simulations. By using the orthogonal array and analyzing the simulation or experiment results, a large amount of data can be obtained with fewer experiment trials. The table and graph of quality characteristic response are obtained through factor effect calculations.

### 3.2. Material Models

#### 3.2.1. Viscosity Model [21,22,23]

The Cross–Castro–Macosko viscosity model is used in this study to describe the viscosity behavior of EMC. It can be expressed by the following equations:

(8) $$\eta \;=\frac{\eta_{0}{\left(\frac{C_{g}}{C_{g}-C}\right)}^{b_{1}-b_{2}C}}{1+{\left(\frac{\eta_{0\dot{\gamma }}}{\tau^{*}}\right)}^{1-n}}$$

(9) $$\eta_{0}=A\cdot \exp\left(\frac{T_{b}}{T}\right)$$

(10) $$T_{b}=\frac{E_{\eta }}{R}$$

C: degree of cure; Cg: degree of cure at gel point; η: viscosity; η_0_: viscosity at zero shear strain; $\dot{\gamma }$: shear strain rate; τ*: critical shear stress; n: power law index; $T_{b}$, $b_{1}$, $b_{2}$, A: viscosity model constants; Eη: time-dependent viscosity activation energy; R: constant of ideal gas.

**Figure 2:**
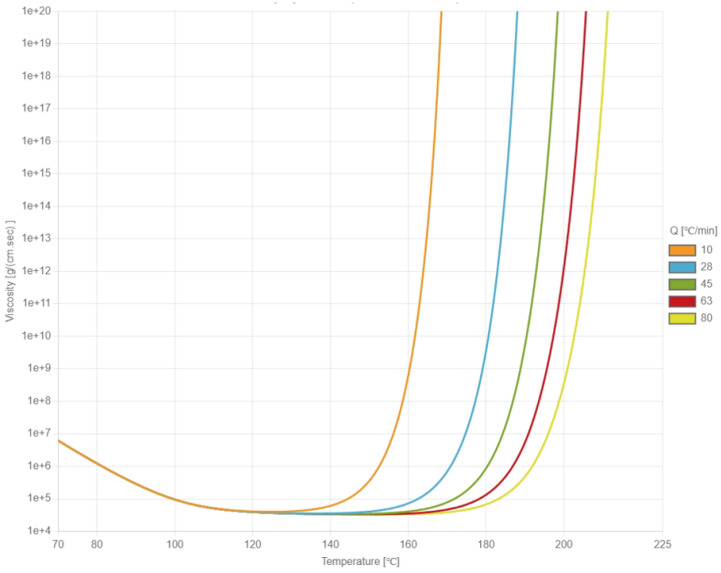
The relationship between viscosity and temperature under different rates of temperature increases.

During the heating process, the viscosity of the EMC initially decreases due to rising temperature and subsequently increases because of the cross-linking reactions. The viscosity behavior of the EMC was measured using an Anton Paar MCR 502 rheometer and fitted to the Cross–Castro–Macosko viscosity model by the laboratory of CoreTech System Co., Ltd. (Hsinchu, Taiwan). In the rheometer experiment, the EMC sample was placed between parallel plates, with a fixed angular frequency applied to the upper plate to induce rotation. The viscosity was determined by monitoring the changes in shear strain rate between the upper and lower plates. Figure 2 illustrates the relationship between viscosity and temperature under different heating rates. The fitted constants of the Cross–Castro–Macosko viscosity model are listed in Table 1.

The Cross–Castro–Macosko model captures the viscosity evolution of thermoset materials by accounting for temperature, shear rate, and degree of cure. The zero-shear viscosity $\eta_{0}$ decreases exponentially with temperature, enhancing flowability during mold filling. The transition of the shear stress $\tau^{*}$ and shear-thinning index n control how rapidly the viscosity decreases with the increasing shear rate. A lower τ or n indicates stronger shear-thinning behavior. The cure-dependent term describes how viscosity sharply increases as the degree of cure C approaches the gel point C, marking the transition to a solid state. The fitting coefficients $b_{1}$ and $b_{2}$ adjust the slope of this increase, especially in later curing stages. Together, these parameters enable the accurate simulation of flow and curing in reactive thermoset resins.

#### 3.2.2. Cure Kinetics Model [24]

Kamal’s cure kinetics model was used to describe the cure behavior of EMC in this study. It can be expressed as the following equations:

(11) $$\dot{C}=\frac{\mathrm{dC}}{\mathrm{dt}}=(K_{a}+K_{b}\cdot C^{m})\cdot {\left(1-C\right)}^{n}$$

(12) $$K_{a}=A\cdot \exp(-\frac{T_{A}}{T})$$

(13) $$K_{b}=B\cdot \exp(-\frac{T_{B}}{T})$$

where $\dot{C}$: curing reaction rate; C: degree of cure; m, n: model constants; A, B: cure reaction frequency factor; K_a_, K_b_: cure reaction rate constant; T_a_, T_b_: activation temperature.

**Figure 3:**
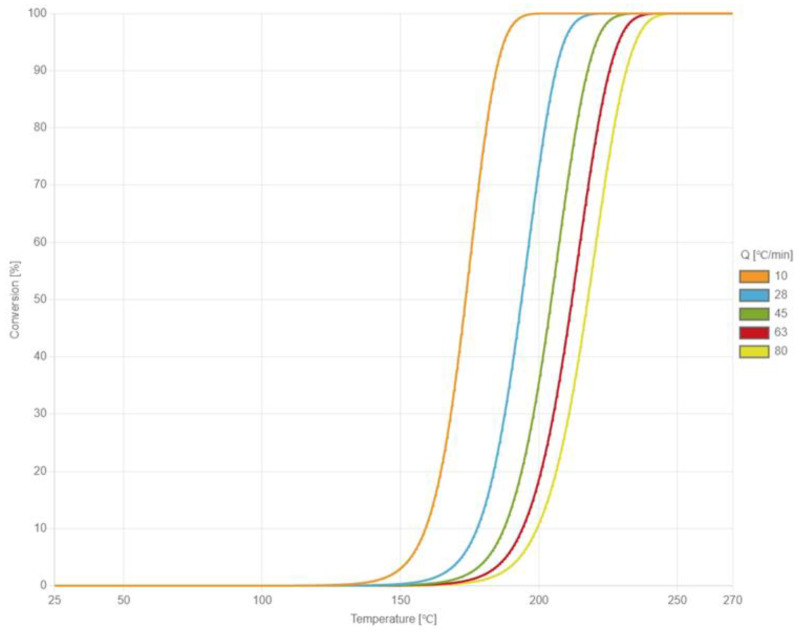
The heat absorption curves of the material as temperature increases under different rates of temperature increase.

When EMC is heated through the B and C stages of cure reaction, cross-linking occurs. Kamal’s cure kinetics model is used to describe the curing behavior of EMC in this study. The cure behavior is measured with a Perkin Elmer DSC 8500 differential scanning calorimetry (DSC) and fitted with Kamal’s cure kinetics model by the laboratory of CoreTech System Co., Ltd. (Hsinchu, Taiwan). In the DSC experiment, the EMC material is divided into two groups and placed in different heating systems. When the test group undergoes phase change or chemical change, endothermic and exothermic phenomena occur, causing the temperature to differ from the control group. Both groups achieve the same temperature through conducting the input and output energy of the control group. Then, they calculate the input or output energy to obtain the curing relationship curve between endothermic and exothermic processes and time. Figure 3 illustrates the heat absorption curve of the material as temperature increases under different rates of temperature increase. The constants of Kamal’s cure kinetics model are shown in Table 2.

#### 3.2.3. P–V–T–C Model [6,7]

The P–V–T–C model is used to describe the specific volume change caused by CTE and chemical shrinkage after the packaging process. It can be expressed as the following equations:

(14) $$\frac{1}{V}=\frac{1}{V_{\mathrm{uncured}}}\cdot (1-C)+\frac{1}{V_{\mathrm{cured}}}\cdot C$$

(15) $$V_{\mathrm{uncured}/\mathrm{cured}\;}=V_{0}\left[1-\alpha \cdot \ln\left(1+\frac{P}{B}\right)\right]$$

(16) $$V_{0}=\left\{\begin{array}{c} b_{1S}+b_{2S}(T-b_{5}),\;\mathrm{if}\;T\;\leq \;T_{\mathrm{trans}}\\ b_{1L}+b_{2L}(T-b_{5}),\;\mathrm{if}\;T\;\geq \;T_{\mathrm{trans}} \end{array}\right.$$

(17) $$B_{p}=\left\{\begin{array}{c} b_{3S}\cdot \exp\left[-b_{4s}\cdot (T-b_{5})\right],\;\mathrm{if}\;T\;\leq \;T_{\mathrm{trans}}\\ b_{3L}\cdot \exp\left[-b_{4L}\cdot (T-b_{5})\right],\;\mathrm{if}\;T\;\leq \;T_{\mathrm{trans}} \end{array}\right.$$

(18) $$T_{\mathrm{trans}}\left(P\right)=b_{5}+b_{6}\cdot P$$

C: degree of cure; V: specific volume; V_0_: specific volume at zero-gauge pressure; α: universal constant; P: pressure; B_p_: pressure sensitivity of the material; b_1S_, b_1L_, b_2S_, b_2L_: coefficient of linear change in specific volume with temperature; b_3S_, b_3L_, b_4S_, b4L: material constants; b_6_: linear increase in Ttrans with pressure; b_5_: transition temperature at zero-gauge pressure.

**Figure 4:**
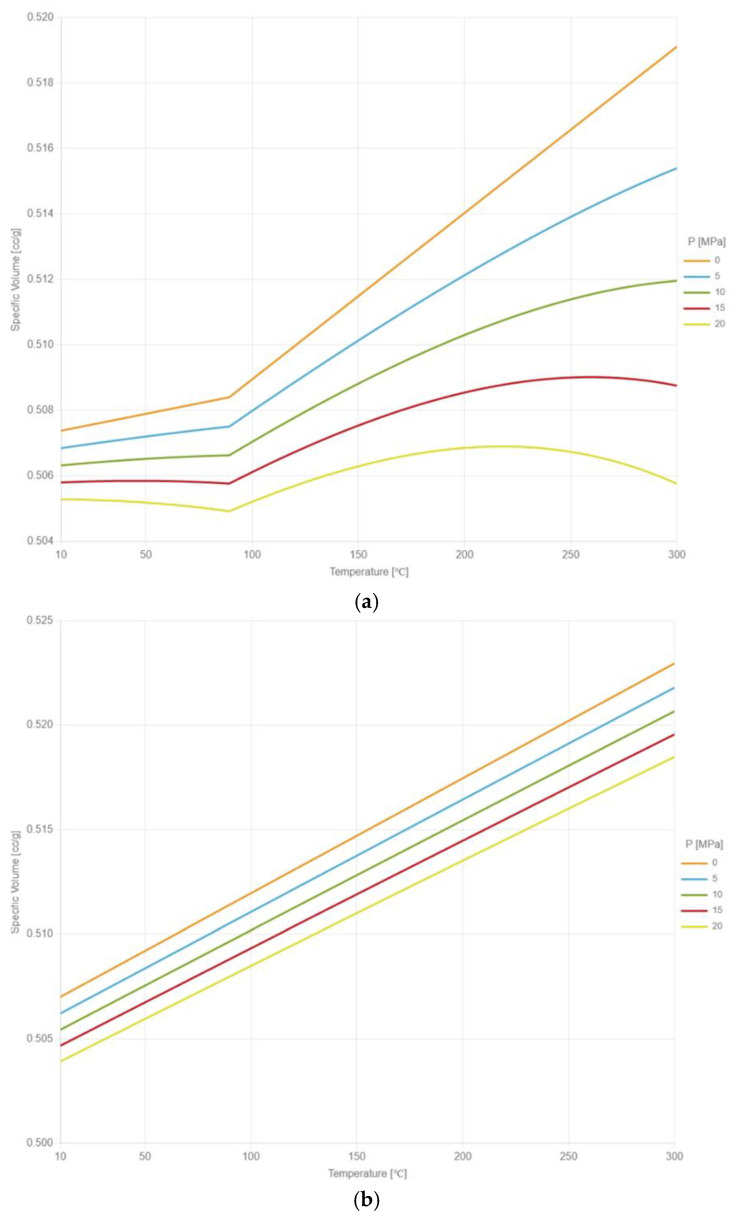
The P–V–T–C relationship: (**a**) fully cured (**b**) uncured.

The measurement data are measured with U-CAN PT-6800 P–V–T–C instrument and fitted with the two-domain modified Tait model by the laboratory of CoreTech System Co., Ltd. (Hsinchu, Taiwan). In the P–V–T–C experiment, curing tests are conducted under various temperature and pressure conditions to establish the relationship between the degree of cure and the specific volume of EMC under different temperature and pressure conditions. Figure 4a illustrates the P–V–T–C behavior of EMC under a fully cured state. The inflection of the curve in Figure 4a corresponds to the glass transition temperature point (Tg point) of the fully cured EMC. Figure 4b illustrates the P–V–T–C behavior of EMC in an uncured state. The experimental data are obtained through incremental temperature measurements. The measurement data of the uncured EMC are obtained below the Tg point of the fully cured EMC with temperature measurements, while the values above the Tg point are obtained by extrapolation. The constants of the two-domain modified Tait model are shown in Table 3.

### 3.3. Mesh Models

**Figure 5:**
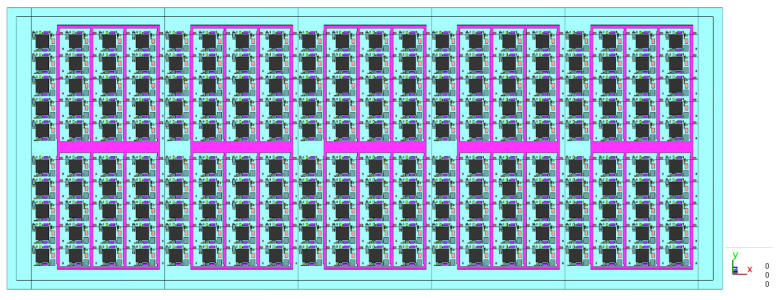
Top view of the whole strip.

The whole strip is not a symmetrical structure, but it can be divided into left and right boundary sections and five identical 1/5 strips, as shown in Figure 5.

#### 3.3.1. The Single-Unit Model

**Figure 6:**
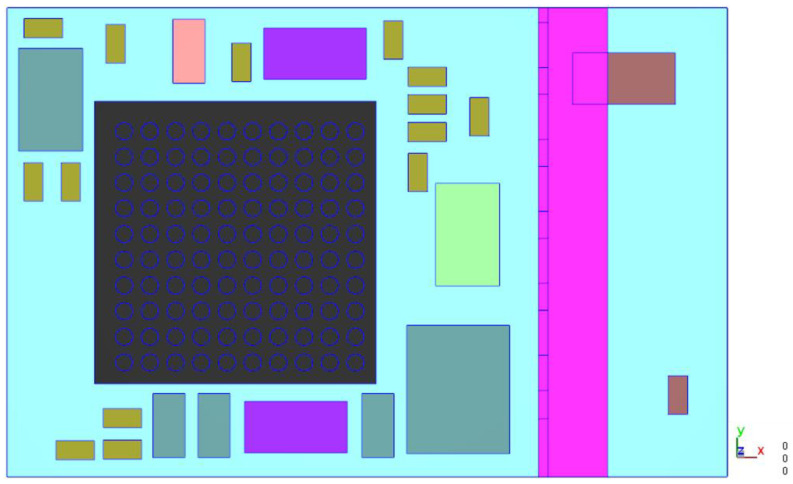
Top view of the single unit model.

This model shown in Figure 6 is used to observe the flow behavior of EMC in a single unit during the compression molding process.

#### 3.3.2. The Leftmost 1/5 Strip Model

**Figure 7:**
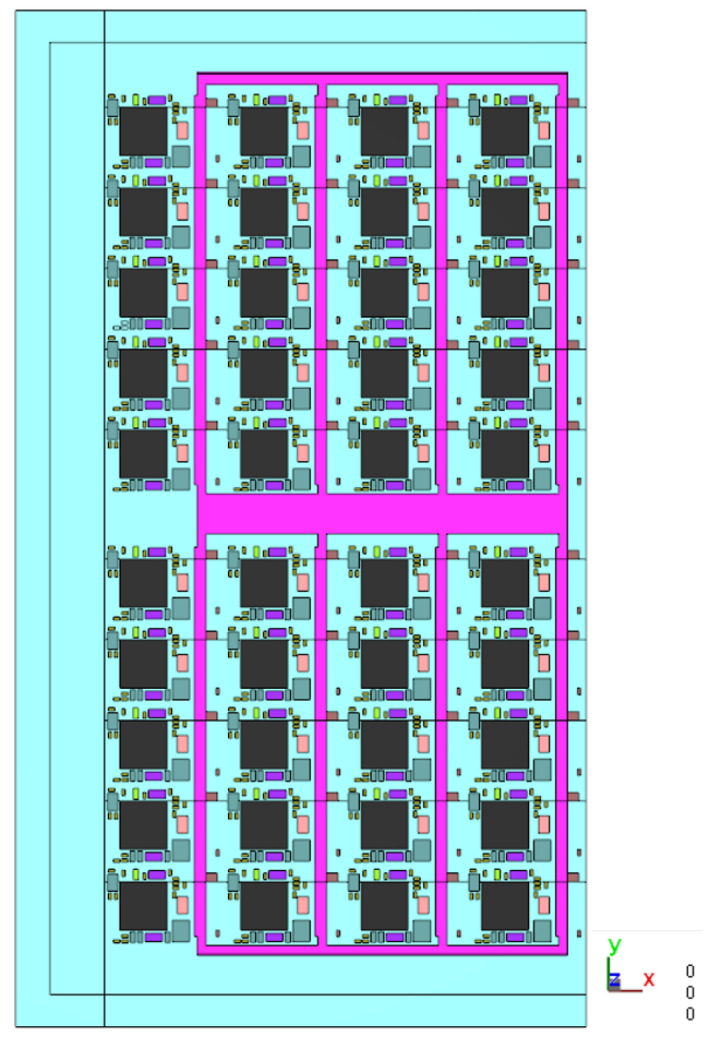
Top view of the leftmost 1/5 strip.

This model shown in Figure 7 includes a left boundary section along with a 1/5 strip. It can be simulated for the vacuum conditions at the junction between the substrate and the fill area. This allows for the observation of the EMC filling behavior in the leftmost column of units.

#### 3.3.3. Mesh Size Setting

Table 4 presents the mesh size, number of meshes, and filling time for models with different meshes.

**Figure 8:**
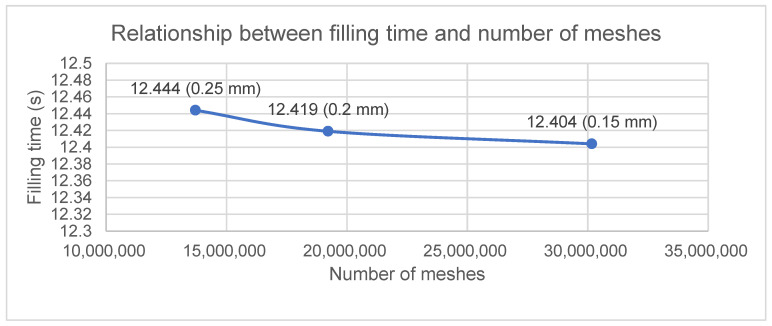
Mesh convergence analysis of the strip.

Figure 8 presents that the mesh size decreases, the results become more convergent.

In the convergence analysis, the results have already converged at a mesh size of 0.15 mm. Using a smaller mesh size would significantly increase the computation time, making it cost-inefficient. Therefore, all subsequent simulations are conducted with a mesh size of 0.15 mm.

**Figure 9:**
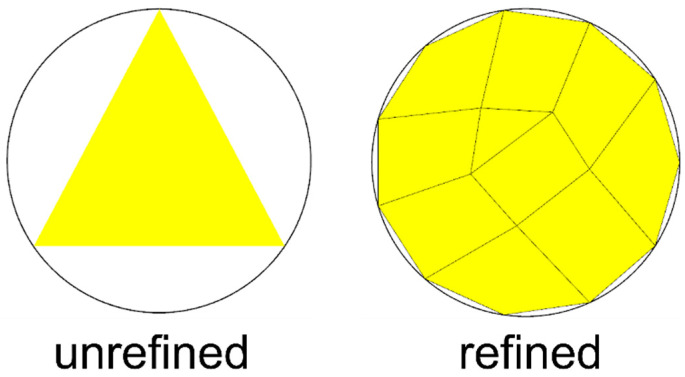
Mesh in the x and y directions of the solder ball.

**Figure 10:**
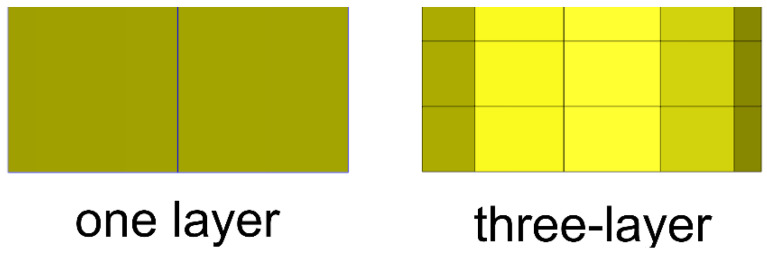
Mesh in the z direction of the solder ball.

The simulation focuses on observing the flow behavior of EMC in the solder ball array between the die and the substrate. Therefore, the mesh is refined in the x and y directions of the solder balls, and three layers of mesh are established on the solder balls, as shown in Figure 9 and Figure 10. According to reference [25], three layers are the minimum number layers that can accurately describe flow behavior.

**Figure 11:**
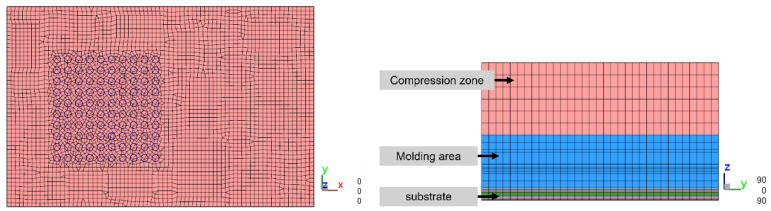
Top view and right view of the single unit mesh model.

Figure 11 Top view and right view of the single unit mesh model below shows the single unit model established with a mesh size of 0.15 mm, and Table 5 presents the number of meshes in the single unit model with a mesh size of 0.15 mm.

**Figure 12:**
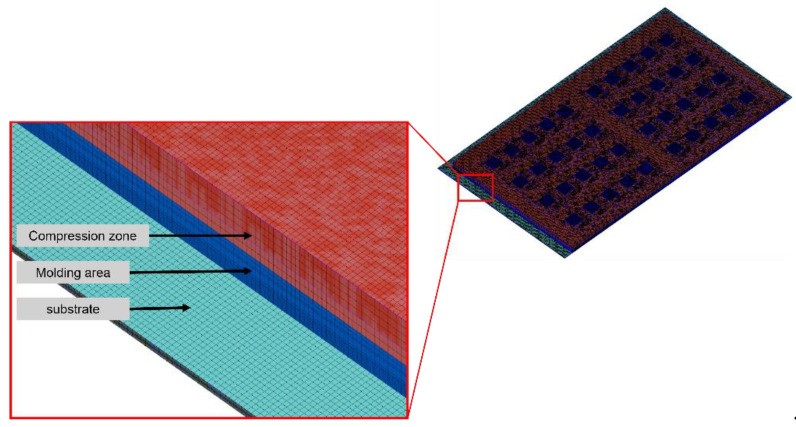
Perspective view of the leftmost 1/5 strip mesh model.

Figure 12 shows the leftmost 1/5 strip model established with a mesh size of 0.15 mm, and Table 6 presents the number of meshes in the leftmost 1/5 strip model with a mesh size of 0.15 mm.

#### 3.3.4. Boundary Conditions

##### Moving Surface

**Figure 13:**
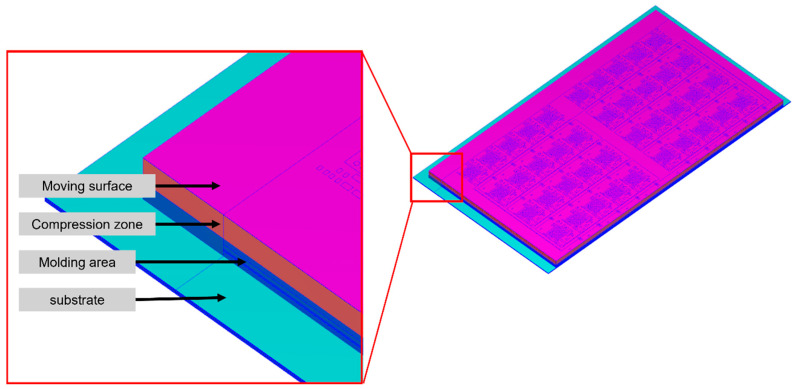
Moving surface.

The moving surface, shown as the green surface in Figure 13, is set on the top of the compression zone. The function of the moving surface is to push the EMC for compression molding filling. The compression direction in the simulations is opposite to that in the actual manufacturing process. Therefore, gravity is set to 980.66 cm/s^2^.

##### Venting Setting

**Figure 14:**
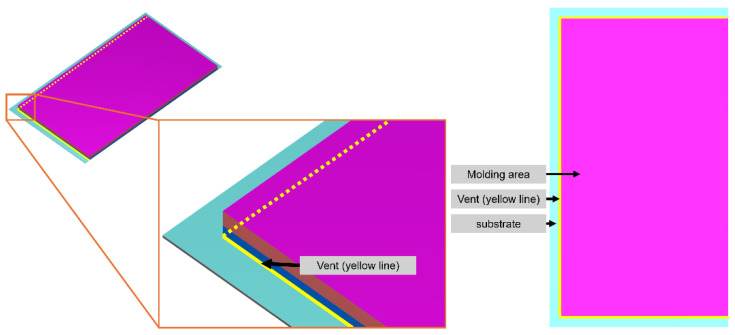
Position of the vent setting.

The venting is set to simulate the vacuum system during the compression molding process. The vent is positioned at the junction between the substrate and the fill area, consistent with the actual manufacturing process, as shown in Figure 14. To expel the air from inside the mold to the outside, the difference of the initial air pressure between the inside and outside of the mold is set. The initial pressure of the air in the mold is set to 0.1 MPa. The initial pressure of the air outside the mold is set to 66.5 Pa because 66.5 Pa is the lowest pressure achievable after vacuuming fabrication.

### 3.4. Materials’ Properties and Process Parameters

Table 7 lists the materials used in the simulation and their properties.

This study uses compression molding for encapsulation, and Table 8 lists the process parameters for the compression molding process. This section primarily observes the flow behavior of EMC, focusing on the preheating and filling processes. Therefore, the compression time in the mold filling analysis is set to only 15 s.

### 3.5. Simulation Results

#### 3.5.1. Results of the Single Unit

Table 9 and Table 10 show the flow behavior of EMC in the single unit in each period. By analyzing the melt front of the EMC, the eventual location of the void formation in each unit is inferred. The actual manufacturing process involving a vacuum system was not known when conducting this simulation, so venting was not set up.

During the mold filling process in the single unit, EMC flows from above the die to beneath the die. Eventually, the melt front surrounds the center of the solder ball array between the die and the substrate, which is the location of the void formation. The eventual void volume can be calculated using the ideal gas equation below.

(19) $$P_{1}V_{1}=P_{2}V_{2}$$

$P_{1}$: the air pressure after vacuuming: 6.65 × 10^−5^ MPa; $V_{1}$: The volume under the die in each unit, excluding the volume of the solder balls; $P_{2}$: The pressure applied to the package by the compression force. $V_{2}$: The volume of the void is to be calculated.

By calculating the above equation, the void area between the die and the substrate in each unit is approximately 11 × 11 µm^2^. According to the simulation result, it can be inferred that the void formation occurs at the center of the solder ball array between the die and the substrate, consistent with the experimental result. However, the reasons why only the leftmost units were detected with voids have not been found. Through discussions with the manufacturer, it is hypothesized that the asymmetry of the strip and vacuuming during the compression molding process causes the void formation in the leftmost units. Therefore, the simulation of the 1/5 strip with venting was conducted.

#### 3.5.2. Results of the 1/5 Strip

Table 11 shows the flow behavior of EMC in the 1/5 strip in each period.

It can be observed that the EMC flow behavior in all columns of units of the 1/5 strip is similar to the simulation results of the single unit, except for the leftmost column of units. In the simulation of the 1/5 strip, in comparison with the EMC flow behavior in other columns of units, the flow on the left side of the dice in the leftmost column of the units is slower. The melt front forms a concave downward curve, resulting in void formations on the left side of the dice in the leftmost column of the units when the area adjacent to the dice in other columns of units is already filled with EMC. According to the simulation of the 1/5 strip, it is hypothesized that the voids adjacent to the left side of the dice in the leftmost column of units are pushed beneath the dice during the compression molding process, resulting in a larger air volume under the dice in the leftmost column of units compared to the other column of units.

#### 3.5.3. Optimization of the Process Parameters by the Taguchi Method

This section will discuss how to reduce the size of voids by adjusting the process parameters. Four process parameter factors are listed in Table 12, and nine experiments were conducted using an L9 orthogonal array.

The Table 13 lists the L9 orthogonal array and the void volume under the dice in the leftmost column of units calculated through the simulation results and ideal gas equation.

**Figure 15:**
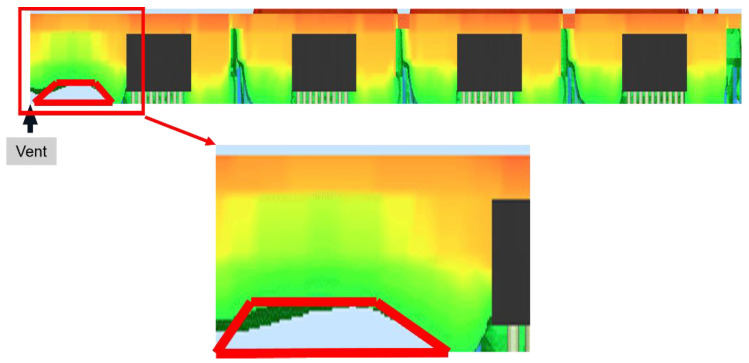
Clipping area of the void.

The quality characteristic is the void size beneath each die in the leftmost column of each unit after compression molding. The air of the void beneath each die after compression molding includes the air adjacent to the left side of the die and the air beneath the die. The final void size is calculated by summing the volumes of these two types of air and substituting them into the ideal gas equation. The volume of the voids adjacent to the left side of the die is calculated by the clipping area of the void, as shown in Figure 15, multiplied by the total length of the 10 dice in a column. The clipping area of the void adjacent to the left side of the die is measured using the distance measurement tool in Moldex3D.2024 R3 The measurement is taken at approximately the 9th second in the filling process, as shown in Figure 15. At the 9th second of the filling time, every area was filled with EMC, except for the area at the left side of the leftmost 1/5 strip and the solder joints array.

Analyze the results in the table above to derive the following response table and response graph of quality characteristics.

**Figure 16:**
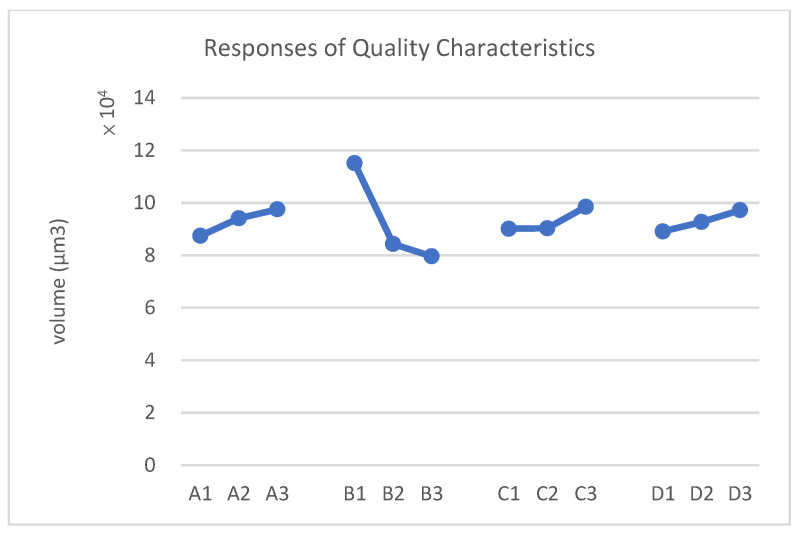
Response graph of quality characteristics.

According to Figure 16 and Table 14, the optimal process parameters are identified as follows: decreasing the compression speed (A1), increasing the compression force (B3), lowering the mold temperature (C1), and reducing the preheating time (D1).

**Figure 17:**
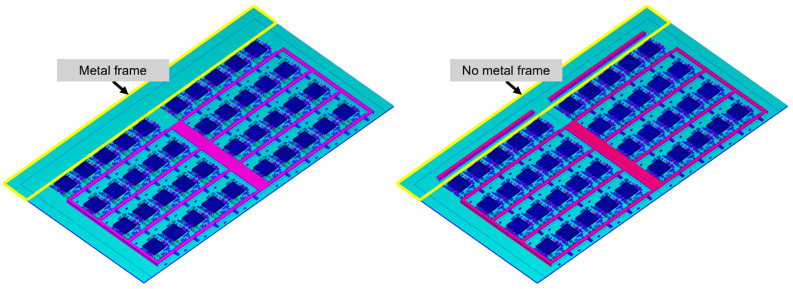
Adding a metal shielding frame to the leftmost side of the strip.

Optimization of adding a shielding metal frame to the leftmost side of the strip by the Taguchi method In the earlier parts of this section, it is hypothesized that the asymmetry of the strip and vacuuming during the compression molding process causes a void formation in the leftmost units. Therefore, whether to add a metal shielding frame to the leftmost side of the strip (Figure 17) is included as a factor in the Taguchi method analysis, along with compression force and compression speed (Table 15).

Because there is one factor with two levels and two factors with three levels, a L18 table is used for the simulation. Table 16 is the L18 table with the calculated results.

Analyze the results in the table above to derive the following response table and response graph of the quality characteristics.

**Figure 18:**
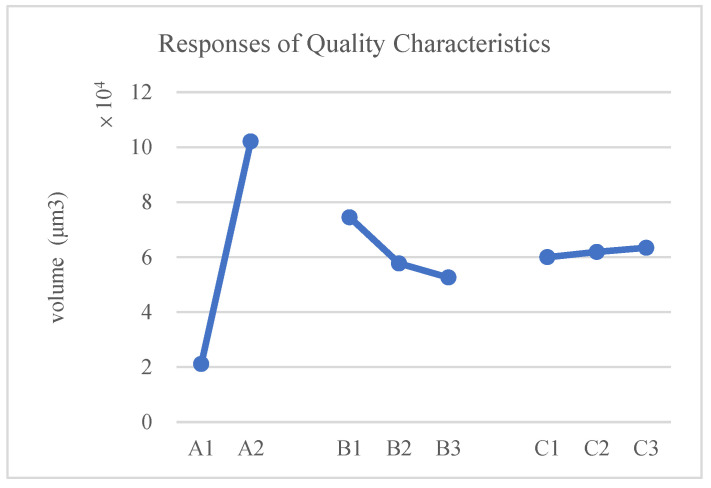
Response graph of quality characteristics.

According to Figure 18 and Table 17, it can be concluded that the asymmetric structure is the cause of the larger void formation in the leftmost column of units. Adding a shielding metal frame to the left side is the most effective method for reducing the size of these voids.

## 4. Conclusions

This study utilizes the Moldex3D 2024 R3 simulation software to conduct mold filling analyses on the SiP packaging considering the vacuum mechanism. The Taguchi method is then utilized to reduce the void size. A simulation method that accurately reflects the actual manufacturing processes is established.

The mold filling analyses, using the viscosity model (cross Castro–Macosko model) and the cure kinetics model (Kamal’s model), effectively simulate the flow behavior of EMC. Through mold filling analyses of a single unit, the 1/5 strip analysis combined with the vacuum mechanism, and the application of the Taguchi method, the following conclusions are drawn:

The void forms between the chip and the substrate, with the EMC flowing downward from the top of the die and trapping air in the center of the solder ball array. Except for each unit in the leftmost column, the void area under the die in the other columns of each unit is approximately 11 × 11 μm^2^.

The vacuum mechanism and the asymmetric geometry of the strip cause a downward concave melt front forming at the left side of the units of the leftmost column during the filling process. Then, the air trapped with the downward concave melt front is eventually pushed beneath the die.

The primary cause of this result is the compression in the molding process, which prevents the air beneath the chip from flowing laterally. As a result, air becomes trapped, forming voids under the chip. The number of voids has already been reduced compared to transfer molding.

According to the Taguchi method analyses, the most effective method to reduce the size of the void is by adding a metal frame to the left side of the strip.

According to the analysis based on the Taguchi method, without altering the geometry of the strip, the factor with the greatest impact on quality characteristics is the compression force of the 1/5 strip, followed by the maximum speed. 

According to the analyses of the Taguchi method, the optimization process parameters without a changing geometric structure of the strip are as follows: (1) compression force: 20 tf; (2) Max. compression speed: 1 mm/s; (3) mold temperature: 160 °C; and (4) pre-heating time: 8 s.

After confirming the size and location of the voids, vent holes can be strategically placed at the corresponding positions to eliminate the voids.

By adding a metal frame on the farthest side, the overall structure becomes more symmetrical and balanced, which facilitates more uniform filling during the flow process. This allows the EMC to flow along the metal frame, resulting in more evenly distributed voids and smaller void sizes.

This study provides a research framework for the heterogeneous packaging project. By accurately predicting void locations and sizes using Moldex3D 2024 R3 and optimizing them through the Taguchi method, it allows for the optimization of model geometry and process parameters before production, thereby enhancing product yield. This approach significantly reduces both cost and time.

## 5. Future Scope

The mold flow analysis results presented in this study provide a valuable reference for future investigations into the flow behavior of EMC polymer materials and the mechanisms of air trap formation. Furthermore, the proposed optimization strategy adding a metal frame to improve flow uniformity demonstrates a practical method for reducing the air trap size. This finding highlights the critical role of geometric design in mitigating void defects. The insights gained from this work can serve as a design guideline for engineers working with polymer-based encapsulation materials, emphasizing the importance of structural symmetry and flow path control in achieving void-free packaging.

This study focuses on the formation of voids during the flow stage, and future research can be extended toward manufacturing-oriented analysis. Specifically, investigating the evolution, size, and final position of voids after the complete manufacturing process, including curing, cooling, and post-processing, would provide a more comprehensive understanding of void behavior and offer deeper insights into defect prevention strategies.

## Figures and Tables

**Table 1:** Parameters of viscosity model.

Model	Parameter	Value	Unit
Cross Castro–Mascosko model	C_g_	0.4	-
	$b_{1}$	15	-
	$b_{2}$	0.1	-
	A	1.5 × 10^−22^	g/(cm-s)
	T_b_	2.5 × 10^4^	K
	n	0.4	-
	$\tau^{*}$	100	dyne/cm^2^

**Table 2:** Parameters of the cure kinetic model.

Model	Parameter	Value	Unit
Kamal’s model	m	0.37	-
	n	0.78	-
	A	0.1	1/s
	B	2 × 10^8^	1/s
	$T_{a}$	1 × 10^4^	K
	$T_{b}$	1.04 × 10^4^	K
	dH	1.8 × 10^8^	erg/g

**Table 3:** Parameters of the P–V–T–C model.

Model	Parameter	Cured	Uncured	Unit
Two-domain modified Tait model	b_1L_	0.51	0.51	cc/g
	b_2L_	5.08 × 10^−5^	5.5 × 10^−5^	cc/(g-K)
	b_3L_	2.51 × 10^9^	2.7 × 10^−9^	dyne/cm^2^
	b_4L_	6.79 × 10^−3^	1.21 × 10^−3^	1/K
	b_1S_	0.51	0.51	cc/g
	b_2S_	1.29 × 10^−5^	5.5 × 10^−5^	cc/(g-K)
	b_3S_	2.51 × 10^9^	2.7 × 10^9^	dyne/cm^2^
	b_4S_	6.58 × 10^−3^	1.22 × 10^−3^	1/K
	b_5_	3.62 × 10^2^	3.23 × 10^−2^	K
	b_6_	1 × 10^−9^	1 × 10^−9^	(cm^2^-K)/dyne

**Table 4:** Mesh convergence analysis of the strip with number of meshes and filling time.

Mesh Size (mm)	0.25	0.2	0.15
Number of meshes	13,709,123	19,220,146	30,168,894
Filling time (s)	12.444	12.419	12.404

**Table 5:** Number of meshes and mesh size of the single unit model.

Mesh Size (mm)	0.15
No. of elements	115,570

**Table 6:** Number of meshes and mesh size of the leftmost 1/5 strip model.

Mesh Size (mm)	0.15
No. of elements	8,636,822

**Table 7:** Materials’ properties.

Component	Material	CTE (ppm/°C)	Young’s Modulus (GPa)	Poisson’s Ratio	Density (g/cm^3^)	Specific Heat (J/g-K)	Thermal Conductivity (W/m-K)
Solder mask	Solder mask	60 (100 °C ↓) 130 (100 °C ↑)	2.4	0.29	1.57	4.86	0.23
Prepreg	Prepreg	11.1	19	0.2	1.5	0.00111	0.7
Core	Core	10.1	23	0.2	1.8	0.001	0.7
Copper	Copper	16.7	110	0.345	8.93	0.385	320
Passive component	Passive components	0.12	106	0.3	5.7	0.28	2.9
Metal frame	Alloy of Cu (60%), Zn (20%) and Ni (20%)	18.7	128	0.319	8.57	0.396	233
Solder joint	Sn	22	50	0.36	7.365	0.444	66.8
Epoxy	EMC	10	20	0.3	1.969	1.07	0.6
Die	Si	2.8	156.9	0.3	2.33	702	124

**Table 8:** Process parameters in mold filling analysis.

Parameter	Value	Unit
Resin temperature	25	°C
Mold temperature	175	°C
Compression time	15	s
Compression speed	2	mm/s
Compression force	18	tf
Pre-heating time	12	s

**Table 9:** Clipping right view of EMC flow behavior in the single unit.

35% (1.5 s)	50% (3.3 s)
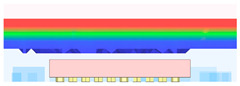	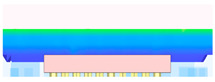
70% (3.8 s)	80% (7.0 s)
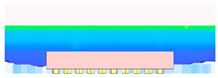	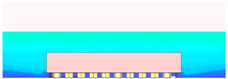

**Table 10:** Bottom view of EMC flow behavior in the single unit.

35% (1.5 s)	50% (3.3 s)
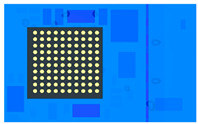	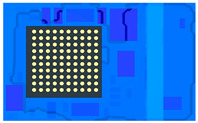
70% (3.8 s)	80% (7.0 s)
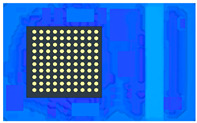	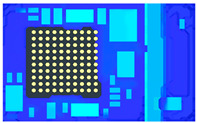
90% (8.1 s)	98% (9.1 s)
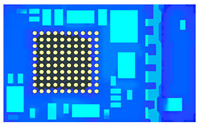	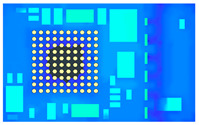

**Table 11:** Clipping front view of the EMC flow behavior in the 1/5 strip under vacuum.

45% (3.1 s)
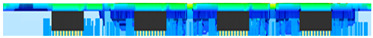
60% (4.7 s)
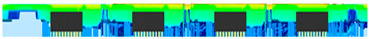
70% (6.4 s)
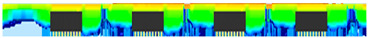
80% (8.6 s)
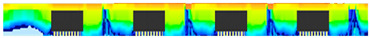

**Table 12:** Table of control factors and level.

	Levels	1	2	3
Factors				
Max. speed (mm/s)		1	2	4
Compression force of the 1/5 strip (tf)		3	3.6	4.2
Mold temperature (°C)		160	175	190
Pre-heating time (s)		8	12	16

**Table 13:** L9 orthogonal array and the void volume results.

Exp.	Max. Speed (mm/s)	Compression Force of the 1/5 Strip (tf)	Mold Temperature (°C)	Pre-Heating Time (s)	Volume (μm^3^)
1	1	3	160	8	10.27 × 10^4^
2	1	3.6	175	12	7.57 × 10^4^
3	1	4.2	190	16	8.37 × 10^4^
4	2	3	175	16	11.77 × 10^4^
5	2	3.6	190	8	8.70 × 10^4^
6	2	4.2	160	12	7.76 × 10^4^
7	4	3	190	12	12.49 × 10^4^
8	4	3.6	160	16	9.02 × 10^4^
9	4	4.2	175	8	7.76 × 10^4^

**Table 14:** Response table of quality characteristics.

Level	Max. Speed (mm/s) (A)	Compression Force of the 1/5 Strip (tf) (B)	Mold Temperature (C)	Pre-Heating Time (D)
1	8.74 × 10^4^	11.51 × 10^4^	9.01 × 10^4^	8.91 × 10^4^
2	9.41 × 10^4^	8.43 × 10^4^	9.03 × 10^4^	9.27 × 10^4^
3	9.75 × 10^4^	7.96 × 10^4^	9.85 × 10^4^	9.72 × 10^4^
Range	1.01 × 10^4^	3.55 × 10^4^	0.84 × 10^4^	0.81 × 10^4^
Rank	2	1	3	4

**Table 15:** Table of control factors and level.

	Levels	1	2	3
Factors				
Shielding metal frame on the leftmost side of the strip		Yes	No	
Compression force of the 1/5 strip (tf)		3	3.6	4.2
Max. speed (mm/s)		1	2	4

**Table 16:** L18 orthogonal array and the void volume results.

	Shielding Metal Frame on the Leftmost Side of the Strip	Compression Force of the 1/5 Strip (tf)	Max. Speed (mm/s)	Volume (μm^3^)
1	Yes	15	1	2.49 × 10^4^
2	Yes	15	2	2.49 × 10^4^
3	Yes	15	4	2.49 × 10^4^
4	Yes	18	1	2.07 × 10^4^
5	Yes	18	2	2.07 × 10^4^
6	Yes	18	4	2.07 × 10^4^
7	Yes	21	1	1.76 × 10^4^
8	Yes	21	2	1.76 × 10^4^
9	Yes	21	4	1.76 × 10^4^
10	No	15	1	12.14 × 10^4^
11	No	15	2	12.60 × 10^4^
12	No	15	4	12.49 × 10^4^
13	No	18	1	8.81 × 10^4^
14	No	18	2	9.28 × 10^4^
15	No	18	4	10.29 × 10^4^
16	No	21	1	8.46 × 10^4^
17	No	21	2	8.91 × 10^4^
18	No	21	4	8.91 × 10^4^

**Table 17:** Response table of the quality characteristics.

Level	Metal Frame on Left (A)	Compression Force (tf) (B)	Max. Speed (mm/s) (C)
1	2.11 × 10^4^	7.45 × 10^4^	6.00 × 10^4^
2	10.21 × 10^4^	5.77 × 10^4^	6.19 × 10^4^
3		5.26 × 10^4^	6.34 × 10^4^
Range	8.10 × 10^4^	2.19 × 10^4^	0.34 × 10^4^
Rank	1	2	3

## Data Availability

The original contributions presented in this study are included in the article. Further inquiries can be directed to the corresponding author.

## Abbreviations and Symbols

*P*	Fluid density
$\overset{⃑}{V}$	Fluid velocity vector
*t*	time
∇	Divergence operator
∇	Gradient operator
*P*	Pressure
$P\overset{⃑}{\overset{⃑}{\delta }}$	Hydrostatic stress
${\overset{⃑}{\overset{⃑}{\sigma }}}_{\mathrm{total}}$	Total stress
$\overset{⃑}{\overset{⃑}{\tau }}$	Extra stress
$\overset{⃑}{g}$	Gravity
$C_{p}$	Specific heat of EMC
*T*	EMC temperature
*k*	Thermal conductivity of EMC
$\dot{\gamma }$	Shear strain rate of EMC
ΔH	The change of EMC enthalpy
$\dot{\alpha }$	Curing rate of EMC
$\overset{⃑}{\overset{⃑}{\sigma }}$	Stress tensor
$\bar{P}$	Mean pressure
$\left[S\right]$	Deviatoric stress tensor
$\overset{⃑}{\overset{⃑}{\varepsilon }}$	Strain tensor
θ	Mean strain
$\left[e\right]$	Deviatoric strain tensor
$R_{\mathrm{ijkl}}(t-\tau )$	Material relaxation modulus components
$\varepsilon_{\mathrm{kl}}\left(0\right)$	Material strain at time zero
*t*	Tiim
τ	Elapsed time
K(t)	Bulk modulus
G(t)	Shear modulus
$\delta_{\mathrm{ij}}$	Kronecker delta function
*e*	Deviatoric part of the strain
*G*(∞)	Shear modulus at full relaxation
*K*(∞)	Bulk modulus at full relaxation
*N*	Number of terms in the Prony series
$\lambda_{i}$	Relaxation time for each term in the Prony series
*C*	Degree of cure
*C_g_*	Degree of cure at gel point
η	Viscosity
$\eta_{0}$	Viscosity when shear strain is zero
$\dot{\gamma }$	Shear strain rate
$\tau^{*}$	Critical shear stress
*n*	Power law index
$b_{1},{\;b}_{2}$, *A*, *T_b_*	Viscosity model constant
$E_{\eta }$	Time-dependent viscosity activation energy
*R*	Ideal gas constant
$\dot{C}$	Cure reaction rate
*C*	Degree of cure
*m*, *n*	Material parameters
$K_{a},K_{b}$	Cure reaction rate constant
$T_{A},T_{B}$	Cure reaction activation energy constant
*A*, *B*	Cure reaction frequency factor
*V*	Specific volume
$V_{0}$	Specific volume at zero-gauge pressure
$B_{p}$	Pressure sensitivity of the material
$b_{1S},b_{1L},b_{2S},b_{2L}$	Coefficient of linear variation of specific volume with temperature
$b_{3S},b_{3L},b_{4s},b_{4L}$	Material parameters
$b_{5}$	Transition temperature at zero gauge pressure
$b_{6}$	Linear increase in transition temperature with pressure
*E*′	Storage modulus
*E*″	Loss modulus
tan⁡δ	Loss factor
ε(t)	Dynamic strain value
$\varepsilon_{0}$	Maximum dynamic strain value
ω	Angular frequency
δ	Phase difference
σ(t)	Dynamic stress value
$\sigma_{0}$	Maximum dynamic stress value
$a_{T}$	Temperature shift factor
$a_{C}$	Conversion shift factor
log*t*	Logarithmic time scale
$\lambda_{i}(T,C)$	Relaxation time related to temperature and degree of cure
*A*_1_, *A*_2_	WLF model empirical constants
$T^{*}$	Critical temperature
$\Delta H_{T}$	Temperature activation energy
*T*_0_	Reference temperature during fitting
$\Delta H_{C}$	Curing activation energy
*R*	Universal gas constant
$T_{g}$	Glass transition temperature
$T_{g0},T_{g1}$	Reference glass transition temperature
γ	Material coefficient
Q	Average quality loss
*n*	*Number of products (n)*
$y_{i}$	Quality characteristic of the i-th product
m	Target value of the quality characteristic
*MSD*	Mean square deviation
${\left(\bar{y}-m\right)}^{2}$	Average eccentricity of the product
$S^{2}$	Standard deviation
$P_{1}$	Initial pressure of the gas after vacuuming but before EMC compression
$P_{2}$	Pressure applied to encapsulation after complete filling
$V_{1}$	Volume under the chip of each unit excluding solder balls
$V_{2}$	Volume to be calculated for the final encapsulation
IC	INTEGRATED circuit
SiP	System-in-package
EMC	Epoxy molding compounds
MUF	Molded underfill
DOE	Design of experiments
DSC	Perkin Elmer DSC 8500 differential scanning calorimetry
Tg point	Glass transition temperature point
